# Fabrication and Characterization of Flexible Solid Polymers Electrolytes for Supercapacitor Application

**DOI:** 10.3390/polym14183837

**Published:** 2022-09-14

**Authors:** Nacer Badi, Azemtsop Manfo Theodore, Saleh A. Alghamdi, Hatem A. Al-Aoh, Abderrahim Lakhouit, Aashis S. Roy, Ayshah S. Alatawi, Alex Ignatiev

**Affiliations:** 1 Department of Physics, Faculty of Science, University of Tabuk, Tabuk 71491, Saudi Arabia; 2 Nanotechnology Research Unit, University of Tabuk, Tabuk 71491, Saudi Arabia; 3 Renewable Energy & Energy Efficiency Center, University of Tabuk, Tabuk 71491, Saudi Arabia; 4 Department of Physics in School of Sciences, Noida International University, Plot 1, Yamuna Expy, Sector 17A, Gautam Budh Nagar 203201, UP, India; 5 Department of Chemistry, Faculty of Science, University of Tabuk, Tabuk 71491, Saudi Arabia; 6 Department of Civil Engineering, Faculty of Engineering, University of Tabuk, Tabuk 71491, Saudi Arabia; 7 Department of Chemistry, S.S. Tegoor Degree College, Gubbi Colony, Kalaburagi 585104, KA, India; 8 Sensor Networks and Cellular Systems Research Center, University of Tabuk, Tabuk 71491, Saudi Arabia; 9 Department of Physics, University of Houston, Houston, TX 77204-5004, USA

**Keywords:** polymer blend, POM, FTIR, EIS, LSV, TNM, GCD, CV, ionic conductivity, transference number, supercapacitor

## Abstract

In this work, solid flexible polymer blend electrolytes (PBE) composed of polyvinyl alcohol (PVA) and polyvinyl pyrrolidone (PVP) with different amounts of sodium thiocyanate (NaSCN) salt mixed in double-distilled water (solvent) are prepared via solution casting method. The obtained films are characterized using several techniques. The study of the surface morphology of the polymer blend salt complex films via the POM technique reveals the presence of amorphous regions due to the NaSCN effect. FTIR spectra studies confirm the complex formation between PVA, PVP, and NaSCN. The addition of 20 wt% NaSCN salt in the composition PVA: PVP (50:50 wt%) polymer blend matrix leads to an increase in the number of charge carriers and thus improves the ionic conductivity. The ionic conductivity of each polymer blend electrolyte was studied using the electrochemical impedance spectroscopy (EIS) method. The highest room temperature ionic conductivity of 8.1 × 10^−5^ S/cm S cm^−1^ is obtained for the composition of PVA: PVP (50:50 wt%) with 20 wt% NaSCN. LSV test shows the optimized ion-conducting polymer blend electrolyte is electrochemically stable up to 1.5 V. TNM analysis reveals that 99% of ions contribute for the conductivity against 1% of electrons only in the highly conductive polymer electrolyte PVA: PVP (50:50 wt%) + 20 wt% NaSCN. A supercapacitor device was fabricated using the optimized ion-conducting polymer blend film and graphene oxide (GO) coated electrodes. The GCD curve clearly reveals the behavior of an ideal capacitor with less Faradic process and low ESR value. The columbic efficiency of the GO-based system is found to be 100%, the GO-based electrode exhibits a specific capacitance of 12.15 F/g and the system delivers the charge for a long duration. The specific capacitance of the solid-state supercapacitor cell was found to be 13.28 F/g via the CV approach close to 14.25 F/g obtained with EIS data at low frequency.

## 1. Introduction

Solid polymer salt complexes are having great worth in diverse electrochemical devices such as fuel cells, supercapacitors, high-energy batteries, display devices, gas sensors, etc., [1,2,3,4]. Because of their excellent interface contact between the electrode and electrolyte, large range of content, and the facilitation of preparation, polymers are highly preferred over crystalline materials. Particularly, polyvinyl alcohol (PVA) is a promising polymer that can dissolve a high amount of several salts to form polymeric electrolyte systems. Polyvinyl pyrrolidone (PVP) is a water-soluble polymer with a high ability for film formation which can be used in many branches of science and technology because of its good properties. To develop new high ion-conducting polymer electrolytes (PEs), highly flexible and strong polymers with good durability are very important for suitable PEs [5]. The ion transport mechanism in the PEs is one of the most important factors to consider when trying to understand how ionic conductivity (referred to hereafter as conductivity) enhancement occurs through the host polymer matrix. Different strategies have been used, including blending two polymers, adding plasticizing agents, and inert inorganic filler materials to PEs [6,7,8,9,10]. The principal benefit of polymeric electrolytes are their mechanical properties, facility of development of thin films of desired sizes, and their ability to form proper electrode-electrode contact [11]. Creating blended polymer systems with improved conductivity is one of the main strategies. In solid polymer electrolyte (SPE) systems, the crystalline nature can be suppressed using a variety of techniques. Thin-film solid-state batteries and electrochemical cells were made using blended PEs. Over the past few years, research on the blending of polymers has revolutionized the sector of pharmacy and industry because of their good properties. Polymer blends can create new compositions by combining existing polymers, producing materials with particular properties for the desired applications [5]. However, the film properties depend upon the miscibility of the blend [12].

Polymer blends are a blend of inorganic and organic. The mixture of two or more polymers is an appropriate approach to enhance both the physical and mechanical properties of PEs including strength and toughness. Physically similar but structurally distinct polymer blends have complexity that is connected by hydrogen bonds, ionic, and dipole interactions. The conductivity can be easily increased by mixing two polymers. To create conventional alternative energy sources, PEs are made using the affordable solution cast technique [13]. 

One effective way to produce new, desirable polymeric materials with high conductivity for a variety of applications is through polymer blending. The qualities of blended polymers are frequently superior to those of individual polymers [14]. PVC/PEO systems (PVC: poly (vinyl chloride); PEO: poly (ethylene oxide); PMMA: poly (methyl methacrylate) and PVC/PMMA based PEs using alkaline metal salts) are described in detail in the literature [14]. When creating polymer films with improved conductivity, some problems can occur, such as high cost, simplicity in film formation, and short battery life. Polymer blends have been produced to address these issues. In general, polymer mixtures ought to be fully soluble in the appropriate solvent, but because of the differences in their chemical structures, miscibility issues can occur at the molecular level [15]. The term “polymer blends” refers to polymer systems created by physically combining two or more copolymers or polymers without a significant amount of chemical reactions taking place between them. The benefits of these types of polymers come from their capacity to be combined with other polymers to create novel compositions, giving rise to materials with particular properties for desired applications. Ionic and dipole interactions, as well as hydrogen bonding, are the most frequent interactions found in blends. However, the miscibility of the blend affects the film’s properties [12]. The high-performance applications for the polymer blends include permeable membranes for separation technology, tissue engineering, and drug delivery [16].

Nam-Soon Choi and his team [17] developed LiClO_4_-doped P (VdF-HFP)-PVAc blend PE which exhibited a conductivity value of about 2.3 × 10^−3^ S cm^−1^ at 25 °C. B. K. Choi et al. [18] fabricated a new polymer blend electrolyte by incorporating LiClO_4_ into the PEO-PAN-EC/BL matrix and the highest value of conductivity value was about 1.2 × 10^−3^ S cm^−1^. Sivadevi and his coworkers [19] showed the ionic conductivity value for NH_4_SCN-doped PVA-PAN blend PE was found to be 2.4 × 10^−3^ S cm^−1^. R. Arunkumar et al. [20] used the solution casting method to create PVC/PBMA-blended PEs doped with LiClO_4_ salt and obtained a better conductivity (1.108 × 10^−5^ S cm^−1^) than that of the pure PVC-based PE (10^−7^ S cm^−1^). Irfan et al. [21] developed SPE based on PVA and PVP doped with sodium fluoride (NaF). The salt incorporation into the polymer blend led to a reduced degree of crystallinity and thus increases its conductivity. The electrolyte system showed a predominance of ionic charges upon electrons and the optical properties of the electrolyte films relied on the content of NaF salt. New solid polymer blend electrolytes were fabricated by Deshmukh et al. [22] using PVA and PVP mixed with various weight percentages of lithium carbonate (Li_2_CO_3_) salt via solution casting technique. As a result, the conductivity has increased with the variation of salt concentration and its maximum value was about 1.15 × 10^−5^ S cm^−1^ for 20 wt% Li_2_CO_3_ and also showed a strong dielectric dispersion due to the Li_2_CO_3_ concentration increase. PVA/PVP/ Li_2_CO_3_ systems seemed auspicious for solid-state batteries. Mohd and his colleagues [23] prepared ion conducting blend PEs based on PVA/PVP with sodium bicarbonate (NaHCO3). The polymer blend electrolytes showed a reduced degree of crystallinity due to an increase in NaHCO_3_ concentration. The obtained films were free-standing with good flexibility and exhibited good mechanical properties. The blend of PVA and PVP expects to create an interchain hydrogen connection between the hydroxyl group of PVA and the carbonyl group of PVP [24]. 

By dissolving inorganic salt in the host lattice, high conductivity and stabilized electrochemical properties are to be obtained from the mixture of PEs. In this work, PVA with its good mechanical properties is mixed with PVP and NaSCN for film preparation. Solid ion-polymer blend electrolytes are developed with different salt contents for supercapacitor device fabrication.

## 2. Materials and Methods

Polyvinyl alcohol (PVA) (Mw 89,000–98,000 g mol^−1^) and polyvinyl propylene (PVP) (Mw: 40,000 g mol^−1^) are used both as host polymers to develop new flexible solid polymer blend electrolytes. Sodium thiocyanate (NaSCN) (Mw: 81.07 g mol^−1^) was utilized as an ion supplier and double-distilled water (DDW) as solvent. All the products were bought from Sigma-Aldrich, Bengaluru (India). The samples were characterized using the Motic (BA 310 model, California, USA) for carrying out the POM method, Perkin-Elmer FTIR spectrometer (model RX1) was used for FTIR measurements, and CH instruments CHI660D (Model 600 series electrochemical analyzer/workstation, USA) was used for EIS measurements.

### 2.1. Polymer Blend Synthesis without Salt

Solid polymer blend electrolyte films were prepared using the solution casting method. An equal amount of 50 wt% PVA and 50 wt% PVP (wt% = weight percentage) are dissolved in DDW separately and placed on a magnetic stirrer for about 4 h. Both solutions were then mixed and stirred for more than 4 h. 

### 2.2. Polymer Blend Synthesis with Salt

The blend solution (PVA-PVP) is then doped with the dissolved solutions of NaSCN with x concentrations (x = 5, 10, 15, 20, 25 wt%). The different mixtures were stirred for 6 h at 50 °C till homogeneous solutions are obtained for each NaSCN composition. All the solutions are poured into Petri dishes at room temperature and placed in a desiccator containing powder silica gels for 2 weeks to remove all the solvent molecules before characterization. After desiccation, solid flexible polymer blend electrolyte films obtained were transparent and were further investigated. The optimized sample is then used for the supercapacitor device as explained in the following sections.

### 2.3. Measurements

All the prepared films of pure PVA, pure PVP, blend PVA/PVP, and PVA/PVP: NaSCN electrolyte samples with different salt content were analyzed via several approaches. The electrochemical characterizations such as EIS, LSV, TNM, and CV analysis are performed using the electrochemical workstation CH-instrument (USA model) over the frequency range 1–0.05 MHz. 

#### 2.3.1. Polarized Optical Microscopy (POM)

The surface morphology of the samples was explored using optical microscopy by Motic (BA 310 model, California, USA) to check the impact of the NaSCN in the PVA: PVP blend.

#### 2.3.2. Fourier Transform Infrared Spectroscopy (FTIR)

The different polymer–ion interactions and specific functional groups present in polymer blend electrolyte samples are determined over the range of 4000–650 cm^−1^ at room temperature using a Perkin-Elmer FTIR spectrometer (model RX1). This technique is also performed to check the complexation between PVA, PVP, and NaSCN.

#### 2.3.3. Electrochemical Impedance Spectroscopy (EIS)

The electrochemical behavior of each sample was studied using the EIS approach which depends on AC potentials frequency. The EIS method is also used to measure the electrical resistance (impedance) between the PE/electrode. This resistance value depends upon the addition of different amounts of NaSCN to the PVA: PVP blend.

##### Conductivity Measurements

**Figure 1:**
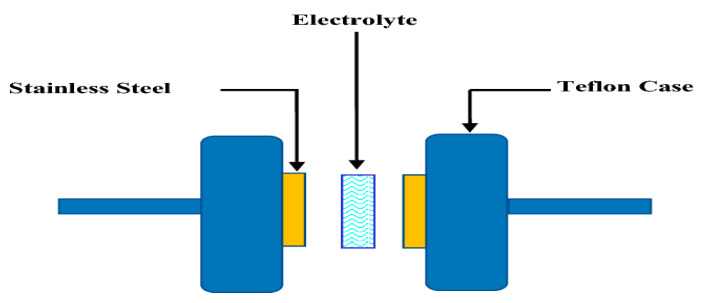
Schematic diagram of cell arrangement for TNM, LSV, and impedance study.

The conductivity (σ) was measured by sandwiching each PE film between two stainless steel electrodes using a sample holder as shown in Figure 1. The conductivity was calculated using the standard formula [25]:

(1) $$\sigma =\frac{t}{R_{b}\times A}$$

where $R_{b}$ is the bulk resistance, A is the section-area (1 × 1 cm^2^) of the film, and t is the thickness of the sample measured with a screw gauge 0–25 mm. 

##### Dielectric Constant

Dielectric constant ($\varepsilon^{*}$) was measured for each sample using Equation (2):

(2) $$\varepsilon^{*}=\varepsilon^{\prime }-j\varepsilon^{\prime \prime }=\frac{1}{j\omega \varepsilon_{0}Z^{*}}$$

The dielectric constant values are calculated from the complex impedance data at 1 kHz frequency.

The real part of the complex permittivity $\left(\varepsilon^{\prime }\right)$ is obtained from Equation (3):

(3) $$\varepsilon^{\prime }=\frac{Z^{\prime \prime }}{(Z^{\prime \prime }{}^{2}+Z^{\prime }{}^{2})\omega C_{0}}$$

where $Z^{\prime }$ and $Z^{\prime \prime }$ are real and imaginary part of impedance spectroscopy ($Z^{*}$), ω is the angular frequency (ω=2πf), $C_{0}=\varepsilon_{0}\frac{A}{d}$ is the capacitance of free space and $\varepsilon_{0}$ permittivity of free space.

##### Carrier Concentration and Mobility

Carrier concentration ($n_{0}$) and mobility (μ) can be determined using Equations (4) and (5) respectively [26]:

(4) $$n_{0}={\left[\frac{\sigma_{0}}{3\varepsilon_{0}{\varepsilon^{\prime }}_{s}\omega_{10\varepsilon }}\right]}^{4}\frac{\varepsilon_{0}{\varepsilon^{\prime }}_{s}KT}{e^{2}d^{2}}$$

where $\sigma_{0}$ is quasi dc conductivity, e elementary charge, K and T are Boltzmann constant and thermodynamic temperature respectively, and the effective dielectric constant is expressed as [26] $\varepsilon^{\prime }\left(\omega_{10\varepsilon }\right)=10{\varepsilon^{\prime }}_{s}$. 

The mobility is then calculated using the following Equation (5) [26]:

(5) $$\mu =\frac{\sigma_{0}}{en_{0}}$$

#### 2.3.4. Linear Speed Voltammetry (LSV)

This approach was carried out to check the breakdown voltage of the optimized polymer blend electrolyte. The working electrode’s current can also be measured using the LSV method as the potential difference between it and a reference electrode is linearly changed over time.

#### 2.3.5. Transference Number Measurements (TNM)

Wagner’s polarization technique is used to evaluate the ionic and electronic contribution of the conductivity. A fixed voltage is given to the sample at a fixed scan rate and the current density is measured over a range of time. The contribution is evaluated by calculating both ion transference number ($t_{ion}$) and electronic transference number ($t_{elec}$) using the following expressions:

(6) $$t_{ion}=1-\frac{I_{ss}}{I_{t}}$$

(7) $$t_{elec}=1-t_{ion}$$

where $I_{ss}$ and $I_{t}$ are the steady state and total currents, respectively.

### 2.4. Synthesis of Electrode Material

Graphene oxide (GO) as electrode material was developed via the exfoliation method [27]. A solution was prepared by mixing H_2_SO_4_ with DDW in the ratio of 10:90 wt%. Platinum cable and graphite bar are connected with electric wires initially linked to a power supply and then placed in the prepared solution. The entire system was protected with a paraffin membrane and received a constant voltage of 4 V that was applied for 5 h to finalize the exfoliation process. The dark solution obtained was entirely ultrasonicated, cleaned, purified, desiccated, and collected. The final product was then placed in the oven at 80 °C. 

### 2.5. Fabrication and Characterization of a Solid-State Supercapacitor

#### 2.5.1. Supercapacitor Fabrication

##### Electrode Fabrication

The highly conductive polymer blend electrolyte and two identical electrodes were used to make the supercapacitor device. The electrodes were prepared using two clean graphite sheets with an area of 1 × 1 cm^2^ as current collectors and doped with an active material (GO). The active material was prepared by dissolving P (VDF-HFP) used as a binder in a small quantity of acetone (solvent) and mixed with GO in a proportion of 10:90 wt% and crushed to form a paste. The graphite sheets were then uniformly coated with the GO paste and further dried in the oven at 80 °C for the whole night. 

##### Supercapacitor Device Assembly

**Figure 2:**
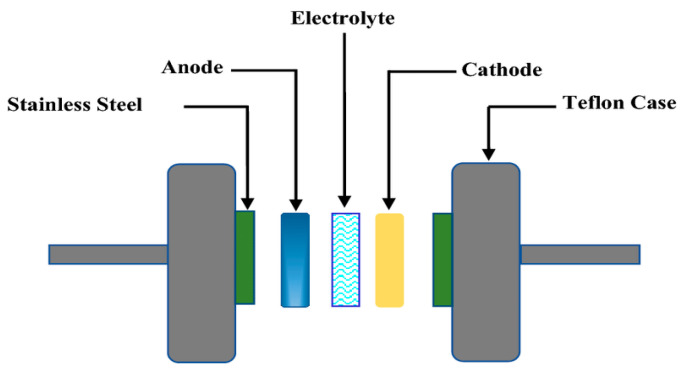
Schematic illustration for the CV and supercapacitor measurements. Adapted from Ref. [28].

Finally, the optimized flexible solid electrolyte was embedded between two identical electrodes to manufacture a supercapacitor. The different components of the supercapacitor device were assembled as shown in Figure 2.

#### 2.5.2. Characterization of the Supercapacitor Device

##### Cyclic Voltammetry

This approach is used to check the electrochemical behavior of the supercapacitor cell at the electrolyte-electrode interface and its performance over a fixed voltage range. The specific capacitance ($C_{sp}$) value of the supercapacitor device is calculated from the graph using the following expression:

(8) $$C_{sp}=2C^{\prime }=2\left(\frac{i}{m\times s}\right)$$

where i is the average current, s is the scan rate, and $C^{\prime }$ is the capacitance of one electrode, and m is the mass of the electrode material.

##### Galvanostatic Charge-Discharge (GCV)

This experiment is experimentally opposite to cyclic voltammetry where the supercapacitor charges and discharges within a range of voltage at a constant applied current. This method reflects the real-world performance of the device. The coulombic efficiency was calculated by using the Equation (9):

(9) $$\eta =\frac{\Delta t_{D}}{\Delta t_{C}}\times 100\%$$

where $\Delta t_{D}$ indicates the discharging time and $\Delta t_{C}$ the charging time.

The specific capacitance of the device was also calculated from the galvanostatic charge/discharge using the Equation (10):

(10) $$C_{sp}=\frac{I}{\left(dV/dt\right)m}$$

where *I* is the discharge current, dV/dt is the slope of the discharge curve, and m is the mass of the electrode material.

##### Low Frequency–Impedance Spectroscopy (IS)

This method is used to measure the specific capacitance at low frequency. The specific capacitance exhibited by the supercapacitor device is then calculated as follows (11):

(11) $$C_{sp}=\frac{-1}{2\pi fZ^{\prime \prime }m}$$

where $Z^{\prime \prime }$ is the low-frequency imaginary part of impedance, m is the active electrode material and f is the frequency.

## 3. Results

### 3.1. Surface Morphology Investigations of Polymer Blend Electrolyte films (POM Analysis)

**Figure 3:**
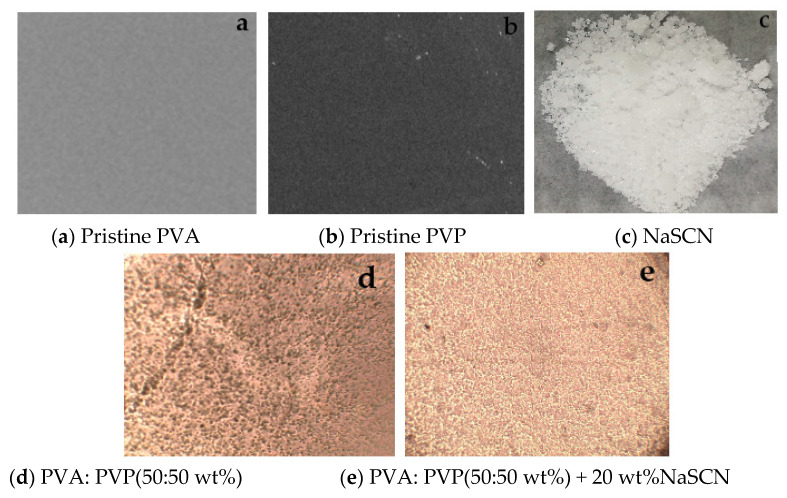
Micrographs of (**a**) pristine PVA film, (**b**) pristine PVP film, (**c**) NaSCN, (**d**) pure polymer blend PVA/PVP film, and (**e**) salt-based polymer blend electrolyte film recorded with (×10 magnifications) and scale bar of 100 μm.

The surface morphologies of pristine PVA, pristine PVP, NaSCN, polymer blend PVA-PVP film, and the optimized PVA-PVP: NaSCN PE samples were recorded and studied (Figure 3). The micrographs of the prepared films appeared to be almost homogeneous and uniform. The surface morphology of pristine PVA film (Figure 3a) and PVP film (Figure 3b) have both a uniform surface morphology with a rather smooth surface. In addition, no features are attributed to a crystalline morphology. Figure 3c shows the NaSCN sample used as a dopant to make a complex system with the polymer blend PVA/PVP. The surface morphology of the pure polymer blend PVA-PVP film and the optimized PVA-PVP: NaSCN film are shown in Figure 3d,e respectively. Both micrographs of polymer blend PVA/PVP films show the degree of homogeneity due to the impact of salt distribution through the blend matrix. The morphology surface of the pure PVA-PVP blend matrix looks uniform and also PVA and PVP are completely miscible. The surface morphology of the pure polymer blend reveals the presence of interconnected micropores. The porosity of the structure is related to the high degree of porosity of PVA which enables adsorption of a large quantity of water while its network structure remains unchanged. PVA can be used as highly porous hydrogels because of the network architecture of interconnected polymer chains that drives molecular diffusion [29]. The porous interconnected structure not only promotes the diffusion of molecules and ions as well within the matrix but also plays the role of filter for the pure PVA-PVP blend which only allows molecules and ions with sizes lesser than the pore size. However, the highly interconnected porous structure of the PVA-PVP blend is favorable for ion transport due to the increase of the O-H and C=O bonds and the amorphous structure of PVP which acts as a plasticizer in the system to enhance the conductivity of the membrane. It is observed that the addition of 20 wt% NaSCN to pure polymer blend has changed the surface morphology due to the salt distribution through the matrix. It can be noticed that the pore size was shrunk due to the penetration of the NaSCN in the cross-linked blend structure (Figure 3e). This reduction in pore size may be related to the salt penetration which promotes better conductivity of Na^+^. However, the crystalline phase remains in the polymer blend matrix.

### 3.2. FTIR Analysis

**Figure 4:**
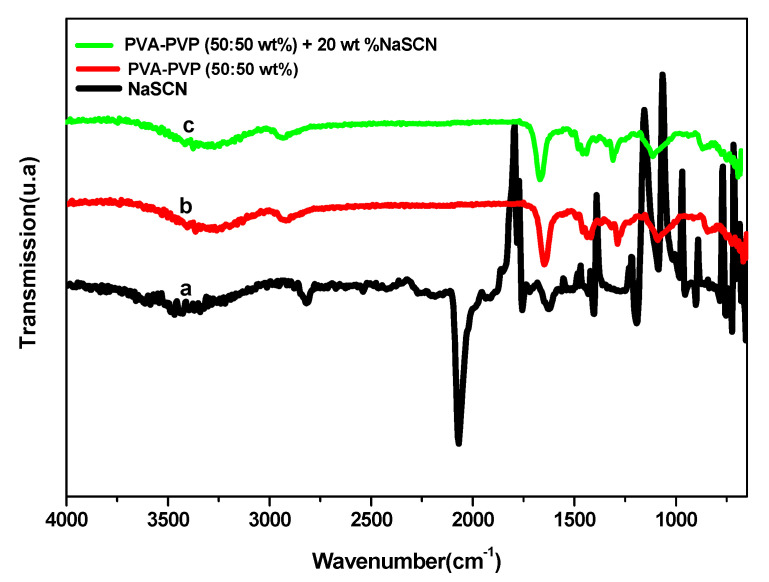
IR spectra of (a) NaSCN, (b) PVA: PVP (50:50 wt%) and (c) PVA: PVP (50:50 wt%) + 20 wt% NaSCN.

The complex formation between the PVA-PVP polymer blend and the NaSCN salt was investigated by FTIR spectroscopy. Figure 4 shows the IR spectra of salt, polymer blend, and the optimized polymer blend electrolyte. Figure 4a reveals the IR spectrum of NaSCN. Some vibration peaks can be clearly observed: at 2821, 2072, 1760, 1438, 1406, 1197, 1089, 907, 728, and 661 cm^−1^. The IR spectrum of polymer blend PVA: PVP (50:50 wt%) in Figure 4b reveals two broad and medium peaks. A vibrational band of the N-H stretch of pure PVP is observed at 3368 and the C-H stretching of pure PVA at 2936 cm^−1^ suggests the presence of amines and carboxylic acids, respectively. The vibration band observed at 1655 cm^−1^ corresponding to C=O stretching of pure PVP is assigned to amides, C=C stretch of pure PVA at 1443 cm^−1^ referred to aromatic compounds, and =C-H bending of pure PVA at 851 cm^−1^ is subjected to alkenes and C-H bending of pure PVP at 832 cm^−1^ are assigned to phenyl groups. Finally, four medium bands of vibration are also seen at 1424, 1294, 1097, and 676 cm^−1^. The bands of vibration of pure PVA and PVP have shifted in the pure PVA-PVP polymer blend with frequency shift. IR spectrum of PVA-PVP-NaSCN is given in Figure 4c and reveals the presence of many vibration bands. The absorption peak observed at 2914 cm^−1^ corresponding to C-H stretching of pure PVA is attributed to alkanes, and C=O stretching of pure PVA at 1652 cm^−1^ suggests the presence of amides of pure PVP, 1465 and 1443 cm^−1^ both of C=C stretching are attributed to aromatic compounds of PVP. New peaks with different intensities occur at 2061, 1294, and 851 cm^−1^ NaSCN-doped PVA-PVP blend films. The additional peaks indicate the interaction between NaSCN and PVA-PVP blend matrix. It should be noted that some peaks present in PVA-PVP-NaSCN (1443, 1424, 1294, 1097 cm^−1^) are also found in the pure PVA-PVP blend. The additional peaks are attributed to NaSCN’s aromatic S-C-N stretching. Due to the attachment of Na+ ions to the C=O present in both pure PVA and pure PVP, the C-H stretching of pure PVA at 2936 cm^−1^ and the C=O stretching of pure PVP at 1655 cm^−1^ have shifted to 2914 and 1652 cm^−1^, respectively, for NaSCN-doped PVA-PVP blend polymer matrix. The change in peak position and intensity observed and the occurrence of the additional peaks confirms the formation of the complex between the PVA: PVP blend and NaSCN. 

### 3.3. AC Impedance Analysis

**Figure 5:**
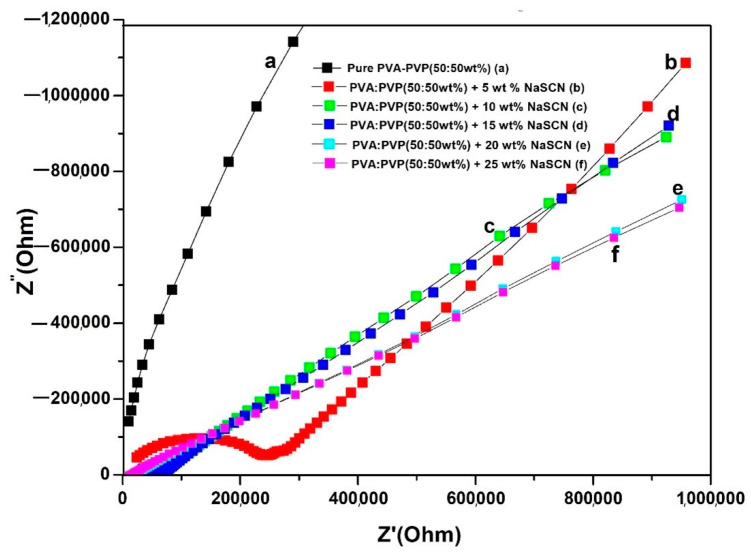
Nyquist plot for polymer blend electrolytes: (a) PVA: PVP (50:50 wt%), (b) PVA:PVP (50:50 wt%) + 5 wt% NaSCN, (c) PVA:PVP (50:50 wt%) + 10 wt% NaSCN, (d) PVA:PVP (50:50 wt%) + 15 wt% NaSCN, (e) PVA:PVP (50:50 wt%) + 20 wt% NaSCN, and (f) PVA: PVP (50:50 wt%) + 25 wt% NaSCN (f) at room temperature.

The electrical behavior of the solid flexible polymer blend electrolytes was investigated via the AC impedance method. Figure 5 shows the Nyquist plots of pure PVA-PVP and PVA-PVP-NaSCN polymer blend electrolytes with different concentrations (5, 10, 15, 20, 25 wt% NaSCN) at room temperature. The graph shows a semicircle in the high-frequency region and a spike in the low-frequency region. The presence of the semi-circle is due to the volume effect of the electrolyte and the spike is caused by the blocking effect of the electrodes [30]. The bulk electrolyte resistance of each sample was determined by the intercept between the semi-circle and the spike. The addition of 5 wt% NaSCN to PVA: PVP (50:50 wt%) has considerably shrunk the diameter of the semi-circle which suggests a decrease in bulk resistance in NaSCN-based polymer blend electrolyte. This reduction may be related to the increase in the number of ionic charges upon the addition of NaSCN. The addition of more salt indicates the appearance of a spike only, which suggests that the PEs’ resistive component exists [31].

#### 3.3.1. Conductivity

The conductivity of each sample was calculated at room temperature using Equation (1) and it is summarized in Table 1. These values are calculated using data obtained in Figure 5.

**Figure 6:**
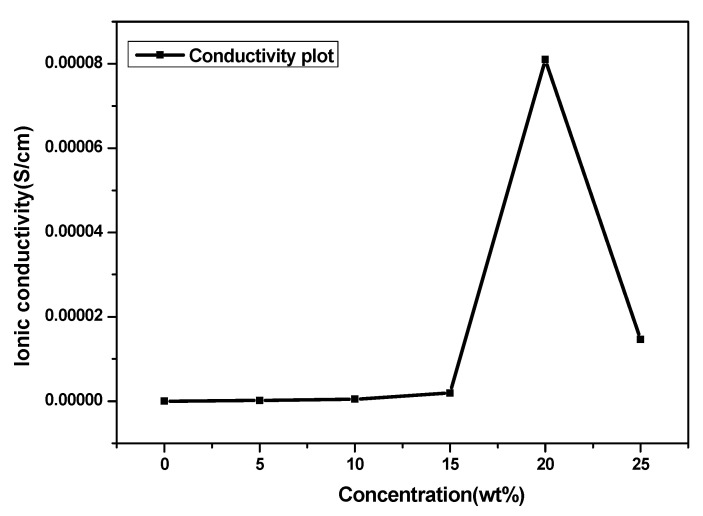
Dependence of conductivity with the concentration of NaSCN in PVA: PVP (50:50 wt%) PE.

It should be noted that the conductivity polymer blend (PVA-PVP) is shown to be superior to that of the pristine polymers. The maximum conductivity was obtained with the composition of PVA: PVP (50:50 wt%) + 20 wt% NaSCN and found to be 8.1 × 10^−5^ S/cm with the lowest bulk resistance. The NaSCN concentration-dependent conductivity is shown in Figure 6.

It is evident that as the amount of NaSCN salt in the polymer blend increases, the conductivity rises. A slight increase in conductivity is observed after doping 15 wt% NaSCN. The addition of salt up to 20 wt% NaSCN has significantly increased the conductivity and further decreases at a higher amount of salt. This strong increase in conductivity is associated with an increase in the number of charge carriers in the PVA-PVP (50:50 wt%) blend matrix and the amorphous nature of the PVA-PVP blend PE which could be due to the interaction between Na^+^ ions and C=O stretching bonds present in pure PVA and PVP. The further decrease in conductivity can be assigned to the increased number of ion aggregates (clusters) in the polymer blend matrix reducing the mobility of charge carriers [32]. The use of NaSCN as an additive material in the PVA: PVP (50:50 wt%) matrix has promoted the transport of Na^+^ and favors the formation of ion-pair complexes. This led to reduced bulk resistance and thus improves the conductivity. 

To sum up, the results obtained with the EIS plots and conductivity plots are well-correlated. EIS data revealed that the addition of NaSCN up to 5 wt% NaSCN showed a significant change in the conductivity trend due to the impact of salt in the polymer blend matrix which influences the conductivity. The incorporation of the salt between 5 and 20 wt% showed a significant reduction of the bulk electrolyte resistance due to NaSCN which improves the conductivity. This result was also confirmed by the data obtained with the conductivity plot, which shows a crucial increase in conductivity between 0 and 20 wt% NaSCN. In addition, the maximum value of the conductivity obtained with the composition 20 wt%NaSCN is also confirmed with the EIS data which indicates the lowest value of the bulk resistance. Finally, the shrinking effect of the semicircle observed in the EIS plot was due to the salt addition and this is confirmed by the increase in conductivity observed in the conductivity plot.

#### 3.3.2. Dielectric Constant

**Figure 7:**
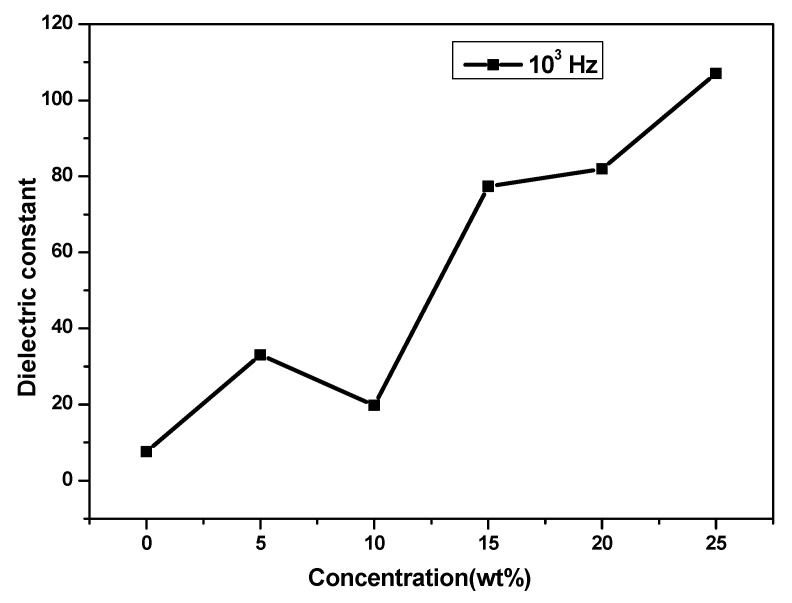
Dielectric constant value with different amounts of salt in PVA: PVP (50:50 wt%) polymer blends at the frequency of 10^3^ Hz.

The variation of the dielectric constant (ε) with the content of NaSCN in PVA-PVP PE is shown in Figure 7. The initial rise in the dielectric constant is brought on by an increase in charge carriers, and the subsequent fall is brought on by the blocking effect of the layer due to the accumulation of ion clusters in the PE. It is seen that the relative permittivity and the conductivity increase with the addition of salt which is correlated to the carrier’s concentration. The value of the relative permittivity with the salt content in PVA-PVP-NaSCN PE was calculated using Equations (2) and (3) and summarized in Table 2.

#### 3.3.3. Charge Carriers’ Concentration and Mobility

**Figure 8:**
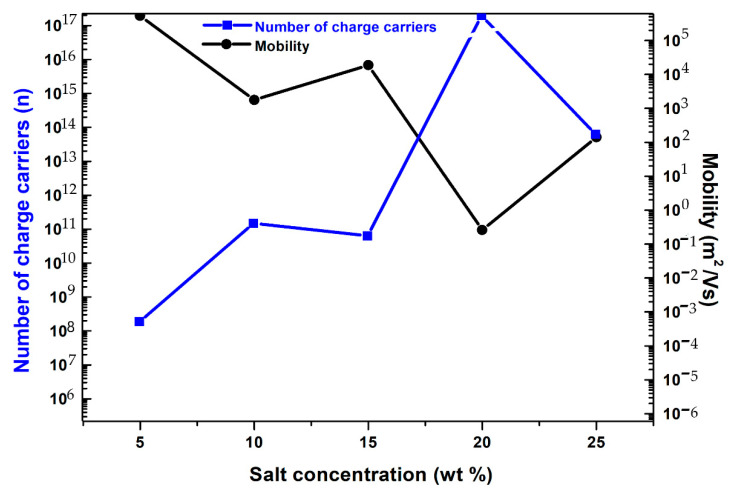
The dependence of the number of charge carriers and mobility on the amount of the NaSCN in the PVA: PVP (50:50 wt%) PE system.

Figure 8 depicts the number of charge carriers and mobility in the PVA-PVP polymer blend system at room temperature as a function of salt concentration. It is observed that the carrier concentration and mobility are opposed to each other. The initial rise in the number of charge carriers upon the addition of 5 wt% NaSCN and further increase up to 20 wt% are due to the augmentation of ion-pair complexes in the polymer-salt complex which increases the conductivity. The dip at 15 wt% is related to the ion agglomerations which do not contribute to the conductivity. A decrease in carrier density at the high content of NaSCN is due to the excess amount of ion clusters in the polymer blend matrix. At a lower amount of salt, the small number of charge carriers in the matrix allows the polymer chain to move more easily, increasing its mobility. At high content, salt in the PVA-PVP (50:50 wt%) matrix acts as an ion source and behaves as a plasticizer at a lower amount improving the chain mobility. However, conductivity is monitored by the carrier concentration. The density of charge carriers and mobility are calculated for different amounts of NaSCN in PE systems at room temperature using Equations (4) and (5) and given in Table 3.

### 3.4. Linear Speed Voltammetry (LSV)

**Figure 9:**
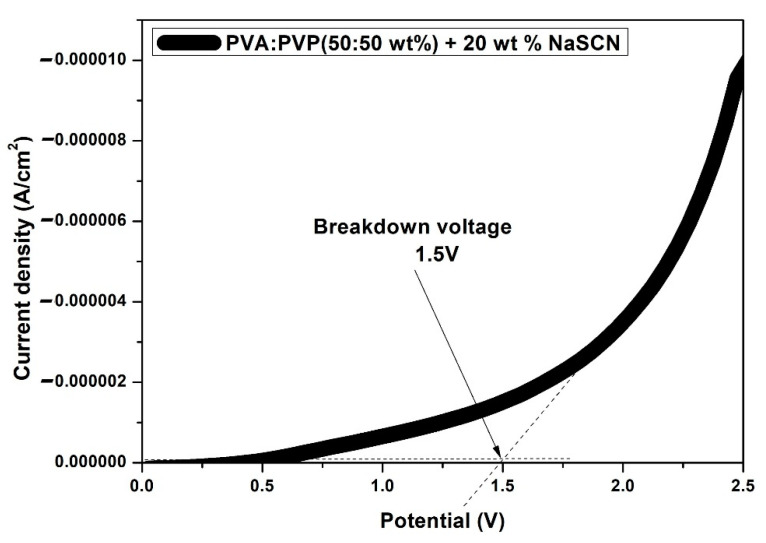
LSV plot for the cell: SS/**SPE**/SS at scan rate 100 mV/s at room temperature.

The decomposition voltage was determined using the prepared solid flexible PE embedded within two stainless steels (SS). Figure 9 shows the LSV plot of the optimized SPE: PVA-PVP (50:50 wt%) + 20 wt% NaCN performed at the scan of 100 mV/s over the range 0–2.5 V. It is clear that no significant current appears over the range 0–1.475 V. Beyond 1.5 V, there is a sharp increase in the current density which is assigned to the decomposition of PE [33]. This assumes that the optimized electrolyte film is stable up to 1.5 V. It is well-known that the typical value of the electrochemical stability potential window (ESPW) for protonic devices is around 1.0 V [34]. Therefore, the optimized PVA-PVP-NaSCN film can be suited for protonic devices.

### 3.5. Transference Number Measurements

**Figure 10:**
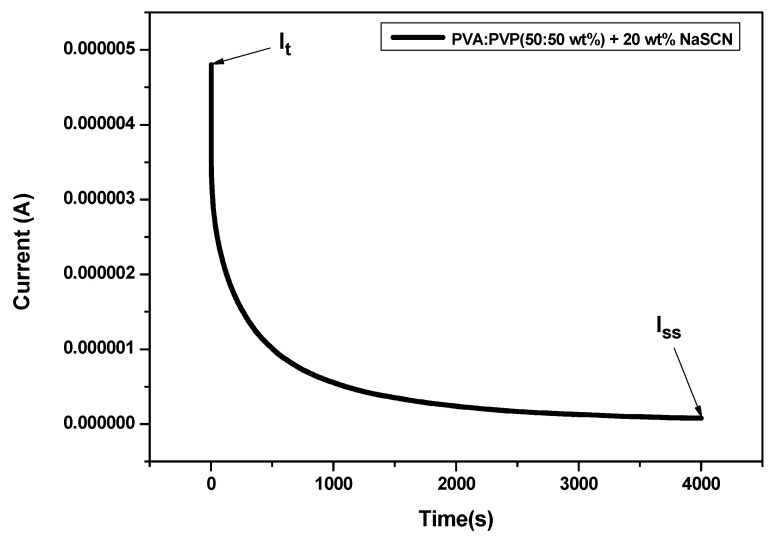
Polarization current vs. Time for the high conductive PE film at applied voltage 80 mV at room temperature.

The Wagner’s polarization method was used to assess how ions and electrons contributed to the total conductivity of the optimized PE. An applied voltage of 0.8 V was given to polarize the cell containing the optimized PE film: PVA-PVP (50:50 wt%) + 20 wt% NaCN. The current was recorded as a function of the time as shown in Figure 10. The curve shows a gradual decrease till its reaches a steady-state current ($I_{ss}=5.34\times 10^{-9}A)$. The total current ($I_{t}=2.92\times 10^{-7}A)$ takes into consideration both ions and electrons at the interface between SS and SPE. The steady-state current confirms the polarization of the cell due to the blocking effect of SS electrodes; however, it allows only the electrons to migrate through [35,36,37]. The ion contribution ($t_{ion}$) and electron contribution ($t_{elec}$) were calculated using Equations (6) and (7) and found to be 0.99 and 0.01 respectively. This result clearly indicates that the conductivity is monitored by ions as the contribution of electrons is very low.

### 3.6. Cyclic Voltammetry

**Figure 11:**
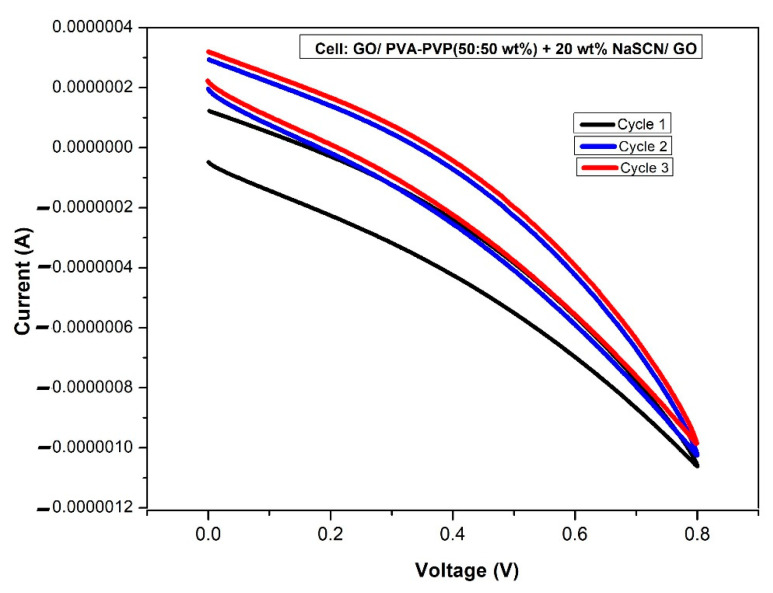
CV plot of the supercapacitor based on the optimized polymer-salt blend electrolyte film recorded at a scan rate 50 mV/s over the potential range 0–0.8 V for the three first cycles.

The performance of the supercapacitor has been evaluated via the CV approach at a scan rate of 50 mV/s over the range of 0–0.8 V for three cycles. Figure 11 shows the capacitive behavior of the fabricated device, which reveals the hysteresis buckle of a supercapacitor. The existence of the supercapacitor was asserted by the presence of no redox peak which suggests the non- faradaic process implied during the charge storage [38]. The CV graph indicates that the total capacitance in the optimal sample is spread over 0–0.8 V and this denotes the importance of the surface area and narrow pore distribution. The supercapacitor gets charged at the electrical double layer due to the induced electric field which holds the ions from the electrolyte and polarizes electrons from the electrode. The CV graph has been recorded to check the device’s performance. The specific capacitance of the supercapacitor device was calculated from the CV graph using Equation (8) and found to be 13.28 F/g. 

### 3.7. Galvanostatic Charge-Discharge (GCV)

**Figure 12:**
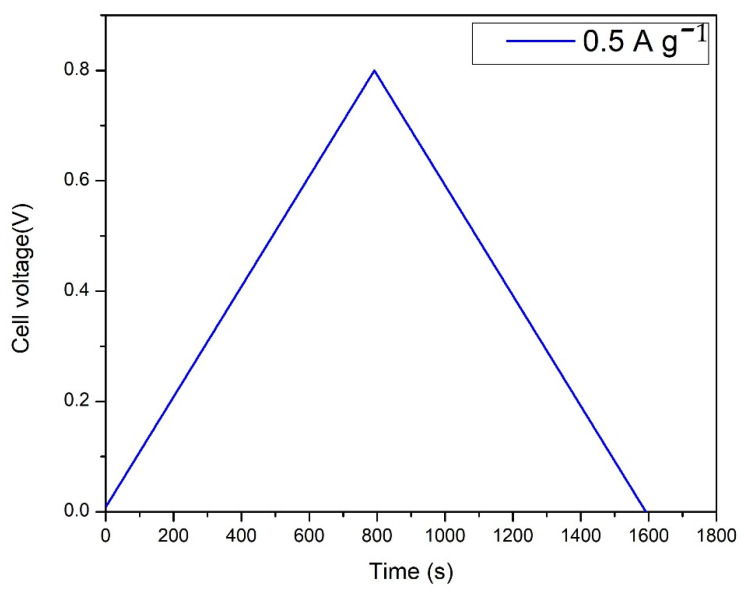
Charge–discharge curve for EDLC with the optimized blend polymer electrolyte at a fixed current density of 0.5 A g^−1^ and at 25 °C.

Figure 12 shows a galvanostatic charge-discharge curve of a supercapacitor made with GO coated on graphene electrodes. Potential is measured with respect to time by applying a fixed current density of 0.5 A g^−1^. It is observed a voltage drop, indicating the presence of internal resistance in the structure. The linear charge/discharge curve clearly reveals the capacitor behavior of the structure with less Faradic process and low ESR value. The coulombic efficiency of the GO-based system is calculated using Equation (9) and found to be 100%, the system delivers the charge for a long duration. The specific capacitance of the device was also calculated from the galvanostatic charge/discharge curve shown in Figure 12 using Equation (10). The calculated specific capacitance value was 12.15 F/g at 0.5 A g^−1^.

### 3.8. Low Frequency–Impedance Spectroscopy (IS)

**Figure 13:**
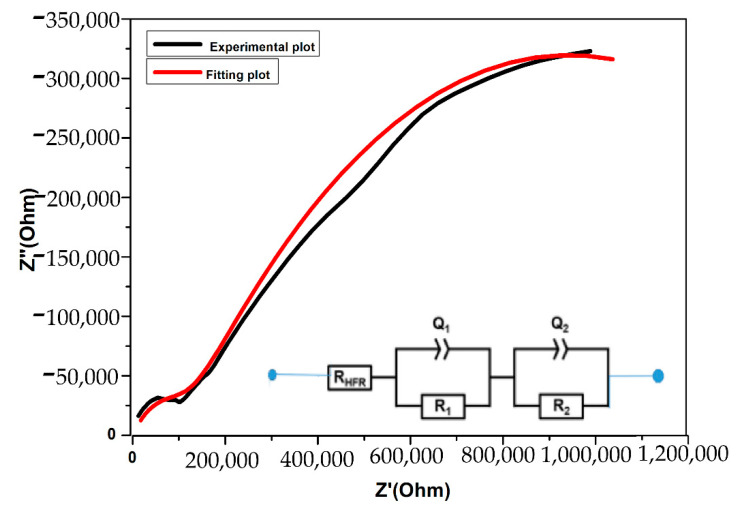
Experimental impedance and EEC fitting plot for the high conducting PE; PVA: PVP (50:50 wt%) + 20 wt%NasCN.

The supercapacitor device’s performance was also evaluated via IS approach at the low-frequency region, described by a capacitive spike indicating the formation of the capacitance double layer and charge accumulation at the contact between the electrode and electrolyte. Figure 13 shows the low-frequency experimental impedance spectroscopy graph of the supercapacitor cell using the maximum solid flexible ion-conducting PE: PVA-PVP (50:50 wt%) + 20 wt% NaCN over the frequency range 1 MHz to 0.05 mHz. The fitting plot was obtained by simulation of the modified Randal’s equivalent circuit [39,40], involving the resistance at high frequency (R_HFR_), the diameter of the first semi-circle (R_1_), which is assigned to the contact resistance between cathode and electrolyte, the diameter of the second semi-circle (R_2_) refers to the charge transfer resistance, Q_1_ and Q_2_ are the constant phase elements associated to R_1_ and R_2_ respectively. R_HFR_ and R_1_ take into consideration the bulk and transport properties. R_2_, Q_1_, Q_2_ feature the insulating and dielectric properties at the electrode and the electrolyte. It is clearly observed that the fitting plot overlaps slightly with the experimental impedance curve. The specific capacitance was calculated at low frequencies (f = 0.03 mHz) using Equation (11), and was found to be 14.25 F/g which is almost close to 13.28 F/g obtained with the CV graph. Table 4 summarizes the value of the different parameters obtained via the EIS data fitted with the equivalent electrical circuit (EEC).

This calculated capacitance value was found to be higher than 1.41 F/g obtained at a rate scan of 10 mV/s with the system PVA: Mg (CF_3_SO_3_)_2_ prepared by Francis et al. [41]. This demonstrates the role of the amorphous structure of PVP in polymer blend electrolyte (PVA-PVP-NaSCN) which could have reinforced the contact between the electrolyte and the active material, limiting the interface resistance and the capacity loss. The low specific capacitance obtained is related to the PE properties although exhibiting good conductivity. However, the specific capacitance strongly depends on the surface area of the electrode material used and also the interface resistance between the active material and the PE. Tough, the specific capacitance can be enhanced by intercalating solidified chemicals into stacked GO sheets to improve the surface area of the electrode as reported by Yi Zhao and his co-workers [42], this study was rather focused on improving the performance of the PE instead of electrode material (GO).

To sum up, the incorporation of PVP and NaSCN into the PVA matrix has not only improved the specific capacitance of the supercapacitor but also significantly increased the number of charge carriers and mobility for the PVA: PVP: NaSCN polymer blend electrolyte. The conductivity of the optimal system: PVA: PVP (50:50 wt%) + 20 wt% NaSCN was found to be one order of magnitude higher than that of the PVA: CS: LiClO_4_ polymer blend system reported by Rathod et al. [43] who obtained a maximum value of 3 × 10^−6^ S/cm for 20 wt%LiCO4 at room temperature without PVP and NaSCN. In another study, the PVA: CS: NH_4_I system exhibited a maximum conductivity of 7.69 × 10^−7^ S/cm for the optimal salt concentration of 40 wt% of NH_4_I [44], which is found to be lower by two orders of magnitude as compared to the PVA: PVP: NaSCN system. This suggests that insertion of NaSCN into the PVA: PVP matrix is favorable for the formation of more ion-dipole interactions and the increase of the amorphous phase due to complexation between Na^+^ cations and the reactive functional groups of PVA: PVP blend present in the PE which augmented the number of mobile species and thus enhances the conductivity. Table 5 shows the ionic conductivity and *t_ion_* values obtained for the recently prepared solid polymer electrolytes and the presently used polymer electrolytes. 

## 4. Conclusions

New solid flexible polymer blend electrolytes based on PVA, PVP, and NaSCN salt as an additive material to improve the conductivity, have been successfully developed via the casting method, and the optimized solid flexible electrolyte was used in a supercapacitor device. FTIR data confirmed the formation of a complex between PVA, PVP, and NaSCN upon the addition of salt (NaSCN) which changed the chemical structure of the whole electrolyte. This result was also verified with POM data which showed a modification of the surface morphology of the matrix due to the salt effect and the formation of the amorphous phase. Impedance spectra demonstrated a significant diminution in bulk resistance due to the salt dispersion which increased the conductivity. The optimized solid flexible polymer blend electrolyte was found to be 8.1 × 10^−5^ S/cm with a concentration of charge carriers of 1.94 × 10^17^ and mobility of 2.61 × 10^−1^ m^2^/Vs. The highly conductive PE is electrochemically stable up to 1.5 V which was prominent for protonic devices since the conventional potential value is 1 V. The conductivity was controlled by ions as the ion transference number was approximately 0.99 for the high ion-conducting polymer blend system: PVA: PVP (50:50 wt%) + 20 wt% NaSCN. The supercapacitor device was fabricated with the optimized PE film and tested. GCD curve reveals an ideal linear charge/discharge behavior with a coulombic efficiency of 100%, and the system delivers the charge for a long duration. The supercapacitor demonstrated a specific capacitance of 13.28 F/g via the CV approach which was close to 14.25 F·g^−1^ obtained with impedance data at low frequency. 

## Figures and Tables

**Table 1:** Calculated conductivity values of blend PEs at room temperature.

Samples	Conductivity (S cm^−1^)
PVA:PVP (50:50 wt%)	4.72 × 10^−9^
PVA:PVP (50:50 wt%) + 5 wt% NaSCN	1.6 × 10^−7^
PVA:PVP (50:50 wt%) + 10 wt% NaSCN	4.2 × 10^−7^
PVA:PVP (50:50 wt%) + 15 wt% NaSCN	1.91 × 10^−6^
PVA:PVP (50:50 wt%) + 20 wt% NaSCN	8.1 × 10^−5^
PVA:PVP (50:50 wt%) + 25 wt% NaSCN	1.46 × 10^−5^

**Table 2:** Dielectric constant value with each amount of NaSCN in polymer blend salt-complex at the frequency 10^3^ Hz.

Samples	Dielectric Constant (ε)
PVA:PVP (50:50 wt%)	7.60
PVA:PVP (50:50 wt%) + 5 wt% NaSCN	32.84
PVA:PVP (50:50 wt%) + 10 wt% NaSCN	19.80
PVA:PVP (50:50 wt%) + 15 wt% NaSCN	77.32
PVA:PVP (50:50 wt%) + 20 wt% NaSCN	81.92
PVA:PVP (50:50 wt%) + 25 wt% NaSCN	107.06

**Table 3:** Values of the number of charge carriers and mobility for different contents of NaSCN in the PVA: PVP (50:50 wt%) polymer salt system.

Salt Composition (wt%)	Number of Charge Carriers (n)	Mobility (m^2^/Vs)
5	1.88 × 10^8^	0.53 × 10^6^
10	1.48 × 10^11^	1.77 × 10^3^
15	6.34 × 10^10^	1.88 × 10^4^
20	1.94 × 10^17^	2.61 × 10^−1^
25	6.28 × 10^13^	1.4×10^−2^

**Table 4:** Values of electrical parameters were recorded from fitting data via Origin Pro 8.0 by using the extrapolation method [39].

Parameter	Value
R_HFR_/kΩ	1
R/MΩ	1.653
R_2_/MΩ	0.1172
Q_1_ (μF)	0.6288
Q_2_ (μF)	0.06045
C_sp_ (F·g^−1^)	14.25

**Table 5:** Summary of the ionic conductivities and *t_ion_* values for some recent solid electrolyte systems and the optimized solid flexible polymer electrolyte (PVA: PVP + 20 wt% NaSCN) prepared in this work.

Samples	Ionic Conductivity (S cm^−1^)	$t_{ion}$ TNM	References
80% PVA:20 wt% PVP	(2.2 ± 1.4) × 10^−7^	-	[14]
PVA/PVP-40 wt% KOH	1.5 ± 1.1 × 10^−4^	-	[14]
P (VdF-HFP)-PVAc/(EC/PC/1MLiClO_4_)	2.3 × 10^−3^	-	[17]
92.5 PVA/7.5 PAN/25% NH_4_SCN	2.4 × 10^−3^	-	[19]
PVA-PVP + 10 wt% NaF	7.50 × 10^−4^	0.89–0.91	[21]
PVA/PVP + 20 wt% Li_2_CO_3_	1.15 × 10^−5^	-	[22]
PVP-PVA/5 wt% NaHCO_3_	1.13 × 10^−5^	-	[23]
50 wt% PVA:50 wt% PVP:30 wt% NH_4_NO_3_	1.41 × 10^−3^		[24]
PVA:CS + 20 wt% LiClO_4_	3 × 10^−6^		[43]
PVA:CS + 40 wt% NH_4_I	7.69 × 10^−7^		[44]
PVA:PVP (50:50 wt%) + 20 wt% NaSCN.	8.1 × 10^−5^	0.99	Present work

## Data Availability

The data presented in this study are available on request from the corresponding author.

## References

[1] Bargav P.B., Mohan V., Sharma M.A.K., Rao V.V.R.N. (2007). Structural and electrical properties of pure and NaBr doped poly (vinyl alcohol) (PVA) polymer electrolyte films for solid-state battery applications. Ionics.

[2] Prajapayti G.K., Roshan R., Gupta P.N. (2010). Effect of plasticizer on ionic transport and dielectric properties of PVA–H3PO4 proton-conducting polymeric electrolytes. J. Phys. Chem. Solids.

[3] Awadhia A., Agarwal S.L. (2007). Structural, thermal and electrical characterization of PVA: DMSO: NH_4_SCN gel electrolytes. Solid State Ion..

[4] Reddy C.V.S., Sharma A.K., Rao V.V.R.N. (2002). Effect of plasticizer on electrical conductivity and cell parameters of PVP+PVA+KClO3 blend polymer electrolyte system. J. Power Sources.

[5] Premalatha M., Vijaya N., Selvasekarapandian S., Selvalakshmi S. (2016). Characterization of blend polymer PVA-PVP complexed with ammonium thiocyanate. Ionics.

[6] Cherng J.Y., Munshi M.Z.A., Owens B.B., Smyrl W.H. (1988). Applications of multivalent ionic conductors to polymeric electrolyte batteries. Solid State Ion..

[7] Yahya M.Z.A., Arof A.K. (2003). Effect of oleic acid plasticizer on chitosan–lithium acetate solid polymer electrolytes. Eur. Polym. J..

[8] Weston J.E., Steele B.C.H. (1982). Effects of inert fillers on the mechanical and electrochemical properties of lithium salt-poly (ethylene oxide) polymer electrolytes. Solid State Ion..

[9] Rajendran S., Sivakumar M., Subadevi R. (2004). Investigations on the effect of various plasticizers in PVA–PMMA solid polymer blend electrolytes. Mater. Lett..

[10] Muthiah M., Chellasamy G., Natarajan R., Subramanian S., Chinnappa S. (2013). Proton conducting polymer electrolytes based on PVdF-PVA with NH_4_NO_3_. J. Polym. Eng..

[11] Subba R.C., Sharmanad A.K., NarasimhaRao V.V.R. (2004). Characterization of a solid state battery based on polyblend of (PVP + PVA + KBr O3) electrolyte. Ionics.

[12] Abdelrazek E.M., Elashmawi I.S., El-Khodary A., Yassin A. (2010). Structural, optical, thermal and electrical studies on PVA/PVP blends filled with lithium bromide. Curr. Appl. Phys..

[13] De Queiroz A.A., Soares D.A., Trzesniak P., Abraham G.A. (2001). Resistive-type humidity sensors based on PVP-Co and PVP-I2 complexes. J. Polym. Sci. B Polym. Phys..

[14] Hatta F.F., Yahya M.Z.A., Ali A.M.M., Subban R.H.Y., Harun K., Mohamad A.A. (2005). Electrical Conductivity Studies on PVA/PVP-KOH Alkaline Solid Polymer Blend Electrolyte. Ionics.

[15] Bash S.K.S., Rao M.C. (2018). Spectroscopic and Electrochemical Properties of (1-x) [PVA/PVP]:[MgCl26H2O] Blend Polymer Electrolyte Films. Int. J. Polym. Sci..

[16] Todd A.M., Otaigbe J.U., Rhoades D., Holland G.P., Cherry B.R., Kotula P.G. (2005). Nanostructured polymer blends: Synthesis and structure. Polymer.

[17] Choi N.-S., Lee Y.-G., Park J.-K., Ko J.-M. (2001). Preparation and electrochemical characteristics of plasticized polymer electrolytes based upon a P (VdF-co-HFP)/PVAc blend. Electrochim. Acta.

[18] Choi B.K., Kim Y.W., Shin H.K. (2000). Ionic conduction in PEO–PAN blend polymer electrolytes. Electrochim. Acta.

[19] Sivadevi S., Selvasekarapandian S., Karthikeyan S., Sanjeeviraja C., Nithya H., Iwai Y., Kawamura J. (2014). Proton-conducting polymer electrolyte based on PVA-PAN blend doped with ammonium thiocyanate. Ionics.

[20] Arunkumar R., Babu R.S., Rani M.U., Kalainathan S. (2017). Effect of PBMA on PVC-based polymer blend electrolytes. J. Appl. Polym. Sci..

[21] Irfan M., Manjunath A., Mahesh S.S., Somashekar R., Demappa T. (2021). Influence of NaF salt doping on electrical and optical properties of PVA/PVP polymer blend electrolyte films for battery application. J. Mater. Sci. Mater. Electron..

[22] Deshmukh K., Ahamed M.B., Polu A.R., Sadasivuni K.K., Pasha S.K.K., Ponnamma D., Mariam A.A.A., Rajendra R.D., Chidambaram K. (2016). Impedance spectroscopy, ionic conductivity and dielectric studies of new Li+ ion conducting polymer blend electrolytes based on biodegradable polymers for solid state battery applications. J. Mater. Sci. Mater. Electron..

[23] Sadiq M., Raza M.M.H., Murtaza T., Zulfequar M., Ali J. (2021). Sodium Ion-Conducting Polyvinylpyrrolidone (PVP)/Polyvinyl Alcohol (PVA) Blend Electrolyte Films. J. Electron. Mater..

[24] Rajeswari N., Selvasekarapandian S., Sanjeeviraja C., Kawamura J., Bahadur S.A. (2014). A study on polymer blend electrolyte based on PVA/PVP with proton salt. Polym. Bull..

[25] Aziz S.B., Abidin Z.H.Z. (2015). Ion-transport study in nanocomposite solid polymer electrolytes based on chitosan: Electrical and dielectric analysis. J. Appl. Polym. Sci..

[26] Schutt H.J., Gerdes E. (1992). Space-charge relaxation in ionicly conducting glasses. II. Free carrier concentration and mobility. J. Non-Cryst. Solids.

[27] Konwar S., Dhapola P.S., Gupta M., Singh R.C., Singh P.K. (2019). High purity graphene oxide using electrochemical synthesis and its application. Macromol. Symp..

[28] Abdulwahid R.T., Aziz S.B., Brza M.A., Kadir M.F.Z., Karim W.O., Hamsan H.M., Asnawi A.S.F.M., Abdullah R.M., Nofal M.M., Dannoun E.M.A. (2021). Electrochemical performance of polymer blend electrolytes based on chitosan: Dextran: Impedance, dielectric properties, and energy storage study. J. Mater. Sci. Mater. Electron..

[29] Shtilman M.I. (2010). Polymers for medicobiological use. Polym. Sci. Ser. A.

[30] Fan L., Dang Z., Nan C.W., Li M. (2002). Thermal, electrical and mechanical properties of plasticized polymer electrolytes based on PEO/ P(VDF-HFP) blends. Electrochim. Acta.

[31] Ramya C.S., Selvasekarapandian S., Savitha T., Hirankumar G., Baskaran R., Bhuvaneswari M.S., Angelo P.C. (2006). Conductivity and thermal behavior of proton conducting polymer electrolyte based on poly (N-vinyl pyrrolidone). Eur. Polym. J..

[32] MacCallum J.R., Tomlin A.S., Vincent C.A. (1986). An investigation of the conducting species in polymer electrolytes. Eur. Polym. J..

[33] Sampath K.L., Christopher S.P.S., Perumal P., Chitra R., Muthukrishnan M. (2019). Synthesis and characterization of biopolymer electrolyte based on tamarind seed polysaccharide, lithium perchlorate and ethylene carbonate for electrochemical applications. Ionics.

[34] Asnawi A.S.F.M., Aziz S.B., Nofal M.M., Yusof Y.M., Brevik I., Hamsan M.H., Brza M.A., Abdulwahid R.T., Kadir M.F.Z. (2020). Metal complex as a novel approach to enhance the amorphous phase and improve the EDLC performance of plasticized proton conducting chitosan-based polymer electrolyte. Membranes.

[35] Devi C., Gellanki J., Pettersson H., Kumar S. (2021). High sodium ionic conductivity in PEO/PVP solid polymer electrolytes with InAs nanowire fillers. Sci. Rep..

[36] Rani M.S.A., Ahmad A., Mohamed N.S. (2017). Influence of nano-sized fumed silica on physicochemical and electrochemical properties of cellulose derivatives-ionic liquid biopolymer electrolytes. Ionics.

[37] Kufian M.Z., Aziz M.F., Shukur M.F., Rahim A.S., Ariffin N.E., Shuhaimi N.E.A., Arof A.K. (2012). PMMA-LiBOB gel electrolyte for application in lithium-ion batteries. Solid State Ion..

[38] Pandey G.P., Kumar Y., Hashmi S.A. (2011). Ionic liquid incorporated PEO based polymer electrolyte for electrical double layer capacitors: A comparative study with lithium and magnesium systems. Solid State Ion..

[39] Xu G.H., Zheng C., Zhang Q., Wei F. (2011). Binder-free activated carbon/carbon nanotube paper electrodes for use in supercapacitors. Nano Res..

[40] Fabio A.D., Giorgi A., Mastragostino M., Soavi F. (2001). Carbon-poly (3-methylthiophene) hybrid supercapacitors. J. Electrochem. Soc..

[41] Francis K.A., Liew C.-W., Ramesh S., Ramesh K., Ramesh S. (2015). Ionic liquid enhanced magnesium-based polymer electrolytes for electrical double-layer capacitors. Ionics.

[42] Zhao Y., Liu J., Wang B., Sha J., Li Y., Zheng D., Amjadipour M., MacLeod J., Motta N. (2017). Supercapacitor Electrodes with Remarkable Specific Capacitance Converted from Hybrid Graphene Oxide/NaCl/Urea Films. ACS Appl. Mater. Interface.

[43] Rathod S.G., Bhajantri R.F., Ravindrachary V., Pujari P.K., Sheela T. (2020). Ionic conductivity and dielectric studies of LiClO_4_ doped poly (vinylalcohol) (PVA)/chitosan (CS) composites. J. Adv. Dielectr..

[44] Marf A.S., Abdullah R.M., Aziz S.B. (2020). Structural, morphological, electrical and electrochemical properties of PVA: CS based proton-conducting polymer blend electrolytes. Membranes.

